# Eucaine Hydrochlorate

**Published:** 1896-09

**Authors:** H. Kiesel

**Affiliations:** Dentist, Berlin, Germany


### EUCAINE HYDROCHLORATE.

BY H. KIESEL, DENTIST, BERLIN, GERMANY.

WHEN one talks or writes of a new local anæsthetic nowadays, it is met with a dubious shake of the head, and either its failure or the report of toxic by-effects that rob it of all its value is awaited. For the list of such remedies is by no means a small one; yet there is none that is always applicable or successful.

Once it was thought that cocaine deserved its place at the head of the list of the local anæsthetics, and it received an enthusiastic welcome. But soon its too frequently severe toxic effects were learned; it was seen that its solutions readily decomposed and, above all, its non-reliability was recognized, since in some cases even large doses were found to have little or no effect, and in other instances the injection of small amounts would be followed by excessive reaction. The author has himself had the experience of an injection of five-hundredths gramme of cocaine (three-fourths grain) causing so violent a reaction that the patient remained in an apparently lifeless condition for an hour and a half. The pulse and the respiration seemed to stop; amyl nitrite was useless, and the patient's life was saved only by the vigorous use of artificial respiration. Palpitation, feelings of apprehension, tiredness, apathy lasting for hours, and even vomiting are not unusual effects of cocaine injections.

In consequence of all this, cocaine was soon almost banished as a means of local anæsthesia from our armamentarium. A substitute for it, on which I am about to report here, will indeed be welcome; I refer to the recently introduced remedy known as eucaine.

Like cocaine, eucaine is the methylester of a benzoylated oxypiperidine carbonic acid, Its constitution is represented by the formula:

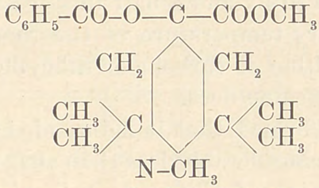

The hydrochlorate of eucaine, which is to replace the hydrochlorate of cocaine, has the following chemical constitution: $C_{19}$-$H_{27}NO_4HCl$.

From a chemical point of view eucaine has an advantage over cocaine in that it is not decomposed by boiling with water; cocaine under similar circumstances dividing into benzoylecgonine and methyl alcohol, and losing its efficacy as a local anæsthetic.

Eucaine, like cocaine, is used in the form of an injection. At first I employed a solution of one to fifteen of sterilized water, using, according to the nature of the operation and the size of the field to be covered, from one to two syringefuls. But I soon found that higher percentages might be employed. I then prepared a solution of one to six and a half, and used that thenceforth in my experiments. Their results were entirely satisfactory.

As regards the advantages of eucaine over cocaine, I note as follows:

1. The heart is in no way influenced by it. In fact I noticed that in very nervous patients, whose pulse had risen to 120 and 130 before the operation, the heart beats became normal and regular very soon after the injection.

2. The anæsthesia is more extensive in area and lasts longer than does that of cocaine. In some of my experiments the analgesia extended even to the muscles. In one case, where an injection was given over the first incisor, there occurred a paralysis of the ala nasi and anæsthesia of the nasal mucous membrane on the right side. The patient declared that her nose felt as if it was stopped up, but the sense of smell was not interfered with.

3. As much as two grammes (thirty grains) of eucaine can be injected without trouble; whilst of cocaine the similarly safe dose is only one-hundredth gramme (one-sixth grain). Thus of a solution of one to six and a half, about fifteen per cent., twelve syringefuls would constitute a maximum dose. Half that quantity would, however, under favorable circumstances be sufficient to painlessly extract all the teeth from a mouth.

4. Solutions of one to six and a half in sterilized water are permanent at the ordinary temperature of the room. They remain clear without the addition of carbolic or salicylic acids and do not become flocculent as cocaine does.

5. Finally, I am informed that it is intended to put eucaine on the market at a price considerably less than that of cocaine.

When we consider the facility of application of eucaine, the certainty of its action, and, above all, its great advantage in the absence of noxious by-effects, it is evident that it will soon become one of our most favored anæsthetic remedies.

I have known the preparation since November, 1895, and have used it exclusively. My results have been such that I have cast aside both ethyl chloride and ethyl bromide entirely.

Who among us has not experienced the excitation and vomiting that so frequently occur in operations done under the bromide of ethyl narcosis? Who among us has not seen ethyl chloride cause excessive secretion of saliva, when extractions of the second and third molars are undertaken? Who has not met cases in which the stream of chloride of ethyl was hindered by the soft parts from reaching the second, not to speak of the third molar? All these unpleasant things may be avoided by the use of eucaine, without in any way interfering with the results that are aimed at.

I use a Pravaz hypodermic syringe and inject the solution about one centimetre in front of the tooth to be extracted. In this way it is rarely difficult to do a painless extraction even of the third molar. In one case, in which on account of anchylosis the syringe could not be advanced far enough—the third lower molar of the left side was to be extracted—I injected from without, in the direction of the diseased tooth. Four or five minutes after the injection the anæsthesia was so great that the extraction could be effected without pain. If, as sometimes does happen, the effect of the drug is not at once apparent, or is not sufficiently marked, it suffices to give a half, or in rare cases a full syringeful additional and both patient and operator will be satisfied with the result. In none of my cases did any of my patients show any disturbance of the system at large.

In a few cases there were swellings of the cheek after the injection, though the solution and the disinfection of the syringe could not be criticised. Thereafter, I cleaned the mucosa thoroughly with cotton soaked in carbolic solution before making the puncture, and I have seen no more of them.

I shall now mention two cases in which I used eucaine which are characteristic, and will enable us to judge of its great applicability in dental practice.

The first was the first one in which I used the drug. It was the extraction of the root of the left upper canine, in consequence of a bet, the condition of which was that the extraction should be done without narcosis. With eucaine it was done painlessly and satisfactorily.

The second case was that of a theological student, who had undergone chloroform narcosis fifteen times in half a year for surgical reasons. I knew that the patient had been operated on under chloroform, but that the narcoses had been so many was purposely concealed from me. The patient had a severe toothache, the cause of which he thought was the second superior molar of the left side, though the second inferior molar of the same side was also painful. The patient could not be convinced of his error, and demanded extraction of the latter under narcosis. To this I finally consented, as the second left inferior molar was already lost. The patient was extremely timid and believed that only very large quantities of ethyl bromide could procure him absolute painlessnesss. He consequently resisted the ethyl bromide action to the utmost. Narcosis occurred only after the administration of twenty-five grammes (five-sixths ounce) of ethyl bromide. I then attempted to extract the tooth; but the patient awoke too early, and the tooth was fractured. In the evening the patient came back with violent pain and demanded that I extract the root. His condition was such that I could not think of another ethyl bromide narcotization. But a syringeful and a half of eucaine sufficed. The next day the patient had become convinced that my opinion was right, and demanded the extraction of the second left superior molar. Again he was given an injection of one and a half syringefuls and again there was the same good effect. The patient assured me that he had felt nothing at all. These good results in extraction, together with similar ones in other operative procedures, induced me to substitute eucaine for the cocaine in my arsenical paste. Here also its action was an excellent one. But seldom were there complaints of slight pain after the introduction of arsenic with eucaine.

Such are my experiences, and they give me the right to ask my colleagues to replace cocaine with eucaine in all cases so as to control my experiments and to extend the usefulness of the drug.

Eucaine is a valuable enrichment of our armamentarium; and will be at least a very dangerous rival to cocaine, as soon as it is better known.—Translated from the *Zahnärztliche Rundschau*, Berlin, April 5, 1896.

Since the publication of the report by dentist Kiesel, it has been found that solutions of eucaine hydrochlorate in the proportion of one part to six and a half parts of sterilized water will, after awhile, separate some of the eucaine. To prepare permanent solutions one part of eucaine should be dissolved in ten parts of sterilized water. Such solutions remain perfectly clear for an indefinite period of time.

